# Short-Term Clinical Results of Ab Interno Trabeculotomy Using the Trabectome with or without Cataract Surgery for Open-Angle Glaucoma Patients of High Intraocular Pressure

**DOI:** 10.1155/2017/8248710

**Published:** 2017-04-17

**Authors:** Handan Akil, Vikas Chopra, Alex S. Huang, Ramya Swamy, Brian A. Francis

**Affiliations:** ^1^Doheny Image Reading Center, Doheny Eye Institute, Los Angeles, CA, USA; ^2^Department of Ophthalmology, David Geffen School of Medicine, Los Angeles, CA, USA

## Abstract

*Purpose*. To assess the safety and efficacy of Trabectome procedure in patients with preoperative intraocular pressure (IOP) of 30 mmHg or higher. *Methods*. All patients who had underwent Trabectome stand-alone or Trabectome combined with phacoemulsification were included. Survival analysis was performed by using Kaplan-Meier, and success was defined as IOP ≤ 21 mmHg, 20% or more IOP reduction from baseline for any two consecutive visits after 3 months, and no secondary glaucoma surgery. *Results*. A total of 49 cases were included with an average age of 66 (range: 13–91). 28 cases had Trabectome stand-alone and 21 cases had Trabectome combined with phacoemulsification. Mean IOP was reduced from a baseline of 35.6 ± 6.3 mmHg to 16.8 ± 3.8 mmHg at 12 months (*p* < 0.01^∗^), while the number of medications was reduced from 3.1 ± 1.3 to 1.8 ± 1.4 (*p* < 0.01^∗^). Survival rate at 12 months was 80%. 9 cases required secondary glaucoma surgery, and 1 case was reported with hypotony at day one, but resolved within one week. *Conclusion*. Trabectome seems to be safe and effective in patients with preoperative IOP of 30 mmHg or greater. Even in this cohort with high preoperative IOP, the end result is a mean IOP in the physiologic range.

## 1. Introduction

Glaucoma is a progressive disease which causes irreversible damage to the optic nerve [1]. The main goal of treatment is to lower intraocular pressure (IOP) to a level which is safe for the optic nerve head. Although trabeculectomy or episcleral aqueous drainage implants demonstrated a permanent IOP reduction, they may have a high risk profile regarding the intraoperative and postoperative complications [2]. This has influenced the development of a less invasive surgical technique, trabeculotomy by internal approach with the Trabectome (NeoMedix Corp., Tustin, CA), which works on the trabecular meshwork and inner wall of Schlemm's canal to reduce outflow resistance [3, 4]. This surgical approach provides a postoperatively stable eye without damaging the conjunctiva and can be further combined with cataract surgery easily with low incidence of intraoperative and postoperative complications.

Results of Trabectome in various types of open-angle glaucoma patients with preoperative IOP of less than 30 mmHg have been shown to be favorable with fewer rates of complication compared to those of traditional trabeculectomy, giving the surgeons hope of an effective and safe treatment option for patients with higher preoperative IOPs [2–4].

The study was conducted to report the success rate of ab interno trabeculotomy within a single-surgeon, single-center cohort of patients with a preoperative IOP of 30 mmHg or higher.

## 2. Patient and Methods

This is a nonrandomized prospective analysis of patients treated by a single experienced surgeon (BAF). The study followed the tenets of the Declaration of Helsinki and the Health Insurance Portability and Accountability Act and had the Institutional Review Board approval. Cohort comparison was studied between patients with open-angle glaucoma-receiving Trabectome combined with phacoemulsification cataract extraction and intraocular lens (IOL) and patients receiving Trabectome alone.

The inclusion criteria for both the combined Trabectome group and Trabectome-alone group were as follows: open-angle glaucoma (as defined by glaucomatous optic nerve appearance with or without glaucomatous visual field damage)—an unobstructed view of the angle, age greater than or equal to 18, a visually significant cataract, and follow-up of at least 2 years. The severity of visual fields was graded according to the Hodapp-Anderson-Parrish (HAP) classification and visual field index (VFI) score [5]. Exclusion criteria were as follows: angle closure, uveitic or neovascular glaucoma, previous glaucoma surgery, and no clear view of the nasal angle.

A total number of 49 eyes of 49 patients were included in the study. Twenty-one eyes underwent combined Trabectome surgery and 28 eyes underwent Trabectome-alone surgery. In each group, patient demographics, preoperative cup-to-disc ratio, preoperative and postoperative visual acuity, IOP, and medications were recorded. Postoperative data at day one and months 1, 3, 6, and 12 were collected.

The surgical procedure has been described in detail elsewhere [2–4]. Briefly, the surgery was performed with the Trabectome® system, including the single-use handpiece with an irrigation-aspiration (I/A) system (Neomedix Inc., Tustin, USA). In combined surgery, the Trabectome surgery was performed prior to phacoemulsification. The head and microscope were tilted to give a gonioscopic view of the angle. The goniosurgical lens (a modified Swann-Jacobs lens) was placed on the cornea to visualize the angle structures. A 1.7 mm keratome was used to create a temporal corneal incision. An ophthalmic viscosurgical device (OVD) was injected to form the anterior chamber. The Trabectome handpiece was inserted and advanced along the meshwork, ablating and removing between 90 and 150 degrees of the nasal trabecular meshwork and inner wall of Schlemm's canal. The power was adjusted up or down depending on the desire to ablate a wider strip of trabecular meshwork or to minimize burning of tissue, respectively. Irrigation and aspiration were then used to remove any remaining blood, viscoelastic, or cellular material.

Postoperative care is varied according to clinical presentation but routinely includes topical steroids four times per day tapered over 8 weeks, topical antibiotics four times per day for 7 days, and pilocarpine 1% three to four times per day tapering over two to eight weeks. Typically, the patients were advised to continue preoperative glaucoma medications after surgery if needed.

The estimated cumulative success rate was obtained by Kaplan-Meier life-table analyses using the following criteria: Kaplan-Meier survival curve of the success of the procedure defined as a decrease in IOP of 20% or more or a decrease in glaucoma medications with no need for additional medications or glaucoma procedures.

## 3. Statistical Analysis

One-way repeated-measures analysis of variance (ANOVA) test was used for the baseline and postoperative values for each group. The difference in IOP and number of antiglaucoma medications between groups were assessed by an unpaired *t*-test. Pearson's *χ*^2^ test was used for subgroup comparison of sex and lens status before surgery. We estimated the cumulative percentages of success as well as the failure rates over time with the Kaplan-Meier method. Statistical significance was assumed for *p* ≤ 0.05.

## 4. Results

**Figure 1:**
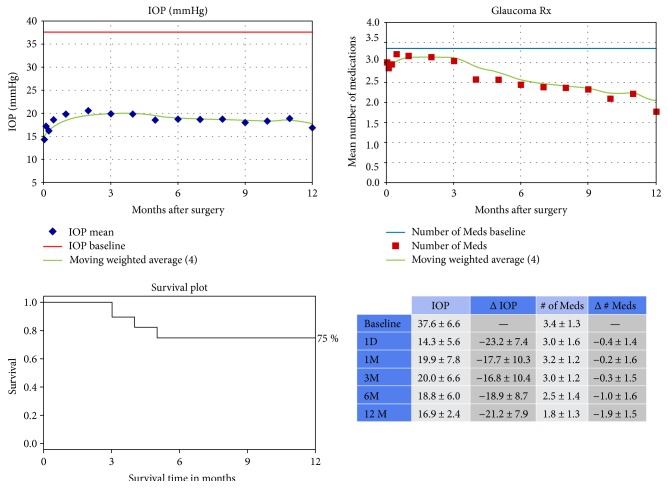
Intraocular pressure (IOP) and number of glaucoma medications data with survival rate over time from all the eyes with IOP > 30 mmHg and having undergone Trabectome surgery with or without cataract extraction. Kaplan-Meier survival curve of the success of the procedure defined as decrease in IOP of 20% or more or a decrease in glaucoma medications with no need for additional medications or glaucoma procedures.

Demographic data and descriptive statistics of 49 cases were included into the study (Table 1). The mean age of the study population was 66 ± 18 and 39% were females. The proportion of Caucasians was higher (63%) and the proportion of African American patients was lower (4%) in the study group. The mean preoperative IOP was 35.6 ± 6.3 mmHg. By postoperative month 12, the average IOP was 16.8 ± 3.8 (55.3% decrease) (*p* < 0.01). The average number of glaucoma medication use was significantly decreased from 3.1 ± 1.3 to 1.8 ± 1.3 at month 12 (*p* < 0.01). Primary open-angle glaucoma (POAG) was the major diagnosis (49%) in the study group and it was followed by pseudoexfoliation glaucoma (24%). Nine patients (18%) needed secondary surgery one year after the surgery and 1 case was reported with hypotony at postoperative 1st day but resolved within one week. The overall survival rate was 80% by postoperative month 12.  Figure 1 shows the IOP and glaucoma medication trend with the survival rate of the procedure during the postoperative follow-up.

Twenty-eight cases had Trabectome-alone surgery and 21 cases had combined Trabectome phacoemulsification surgery. There were some statistically significant differences found between the two groups. The preoperative IOP was significantly lower in the combined Trabectome group (33.0 ± 4.9 mmHg) compared to that in the Trabectome-alone group (37.6 ± 6.6 mmHg) (*p* = 0.01). The Trabectome only group had a better preoperative visual acuity, which reflects the presence of the cataract in the combined Trabectome group. The mean age of the combined Trabectome group was 72 ± 17 and 57% were female. However, the mean age of the Trabectome-alone group was 62 ± 18 and 75% were male (*p* = 0.06). The study reported a higher proportion of Caucasians and lower proportion of Asian patients in both groups. The Trabectome-alone group showed a higher proportion of severe visual field defects compared to the combined Trabectome group. Tables 2 and 3 give the demographic data of each group.

## 5. Combined Trabectome Group

**Figure 2:**
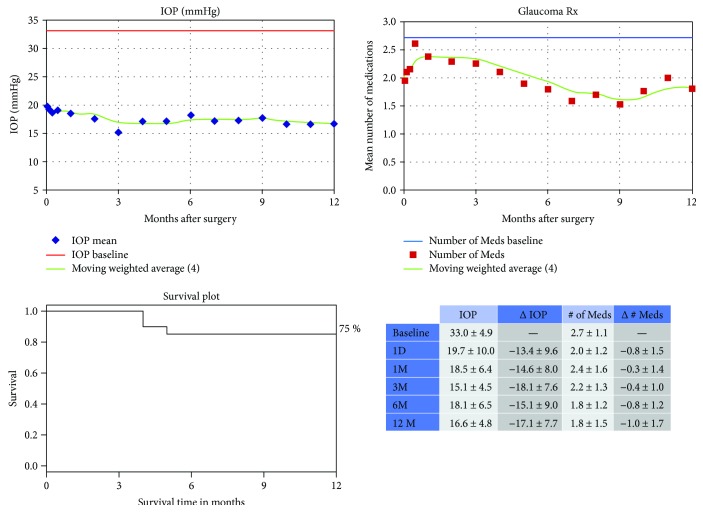
Intraocular pressure (IOP) and number of glaucoma medications data with survival rate over time from the eyes with IOP > 30 mmHg and having undergone combined Trabectome surgery. Kaplan-Meier survival curve of the success of the procedure defined as decrease in IOP of 20% or more or a decrease in glaucoma medications with no need for additional medications or glaucoma procedures.

The mean preoperative IOP was 33.0 ± 4.9 mmHg (Figure 2) and by postoperative month 1, it has dropped to 18.5 ± 6.4 (44.2% decrease). By postoperative month 12, the average IOP was even lower at 16.6 ± 4.8 (51.8% decrease) (*p* < 0.01). Figure 2 shows the IOP and glaucoma medication trend with the survival rate during the postoperative follow-up. The average number of glaucoma medications use in the group was 2.7 ± 1.1. By postoperative month 12, it has significantly decreased to 1.8 ± 1.5 (*p* < 0.01). Survival rate at 12 months of follow-up was 86%. One eye (5%) needed secondary surgery to control IOP one year after the surgery. Hypotony, aqueous misdirection, wound leak, and postoperative infection were not reported in any of the patients. There was no clinically significant bleeding which may require intervention.

## 6. Trabectome-Alone Group

**Figure 3:**
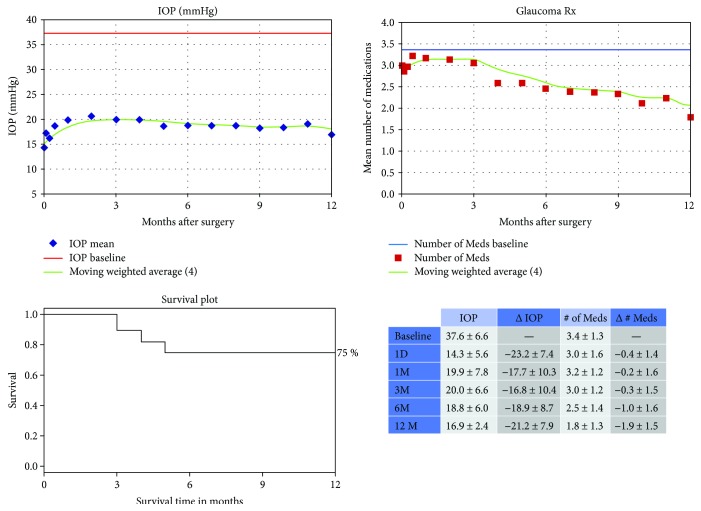
Intraocular pressure (IOP) and number of glaucoma medications data over time from the eyes with IOP> 30 mmHg and having undergone Trabectome-alone surgery. Kaplan-Meier survival curve of the success of the procedure defined as decrease in IOP of 20% or more or a decrease in glaucoma medications with no need for additional medications or glaucoma procedures.

The mean preoperative IOP was 37.6 ± 6.6 mmHg (Figure 3) and on postoperative day 1, it has decreased to 14.3 ± 5.6 mmHg (61.7% decrease). But by postoperative month 1, IOP increased to 19.9 ± 7.8 (47.1% decrease). By postoperative month 12, the IOP was stable at 16.9 ± 2.4 (56.9% decrease). The average number of glaucoma medications used in the group was 3.4 ± 1.3. By postoperative month 12, it has significantly decreased to 1.8 ± 1.3 (*p* < 0.01). Figure 3 shows the IOP and glaucoma medication trend with the survival rate during the postoperative follow-up. Eight cases required secondary surgery. Hypotony (IOP < 5 mmHg) at postoperative day one was observed in one patient (4%) and resolved later.

## 7. Discussion

The Trabectome seems to be a favorable method of minimal invasive glaucoma surgery with or without cataract surgery in patients with preoperative IOP of 30 mmHg or greater. The current data also suggests the effectiveness of Trabectome-alone surgery in reducing IOP and postoperative number of medications compared to combined Trabectome surgery.

The baseline IOP in our study was 33.0 ± 4.9 mmHg in the combined Trabectome group and 37.6 ± 6.6 in the Trabectome-alone group which is higher than the values in the studies by Francis [3] (22 mmHg). Minckler et al. [4] (25.7 mmHg), Jea et al. [6] (28.1 mmHg), or Trabectome-alone surgery significantly reduced the postoperative IOP in our study patients as well as combined Trabectome surgery. The IOPs at 1 year after surgery were significantly reduced from baseline to mid teens (16.9 ± 2.4 mmHg and 16.6 ± 4.8 mmHg, resp.) which is similar to those previously reported [2–6]. These results suggest that Trabectome surgery with or without cataract extraction may offer a clinically useful control on IOP levels. Some studies reported IOPs as 16.1 mmHg [4], 17.4 mmHg [6], and 16.6 mmHg [7] after 1 year of Trabectome surgery. Moreover, in this study, the number of medications were significantly reduced after both surgeries similar to other studies [3, 4, 8]. The success rate after Trabectome surgery has been reported to be about 30%–50% in the literature [2–4, 6–8]. In our study, the success rate for IOP decrease was 55% in the overall study population, 51.8% in the combined group, and 56.8% in the Trabectome-alone group. Mizoguchi et al. [9] reported that their Trabectome failure rate was higher in the eyes with a preoperative IOP <18 mmHg and lower in those with a preoperative IOP of 18–22 mmHg, and they concluded that the results of Trabectome surgery may differ according to baseline IOP. Although the relationship of the surgical success and preoperative IOP level has not been established yet, our study showed that Trabectome surgery can be effective and safe at baseline IOP levels around 35.6 (±6.3) mmHg. Markedly high and low baseline IOPs have been reported as risk factors for poor surgical outcomes [6, 7].

The current study had a control group of glaucoma patients having Trabectome surgery alone; therefore, it was possible to determine to what extent Trabectome trabeculotomy or cataract extraction contributed to the lowering of IOP and medications. The IOP was lowered by 17.7 ± 7.7 mmHg (51.8% decrease) in the combined Trabectome group and 21.2 ± 7.9 mmHg (56.9% decrease) in the Trabectome-alone group by postoperative month 12. It has been generally suggested that phacoemulsification cataract extraction alone may lower IOP in glaucoma patients as well as in nonglaucomatous individuals, with the amount of 2–4 mmHg [10, 11]. Our study showed that there is a decrease to the normal physiologic level in IOP after a Trabectome procedure. Although a higher proportion of IOP decrease was reported in the Trabectome-alone group, it may be caused by higher baseline IOP levels compared to that in the combined Trabectome group.

In a prospective interventional study [12], patients with open-angle glaucoma underwent combined Trabectome surgery. Mean preoperative IOP was 20.0 ± 6.3 mmHg, and mean postoperative IOP was 15.5 ± 2.9 mmHg, with a 1.4 ± 1.3 mean number of glaucoma medications after one year of follow-up. Nine patients needed additional glaucoma procedures.

Another study with a large number of case series evaluated the outcomes of Trabectome-alone versus combined procedures with phacoemulsification [4]. At 24 months, IOP decreased by 40% from 25.7 ± 7.7 mmHg preoperatively to 16.6 ± 4.0 mmHg in the Trabectome-alone group compared to 30% from 20.0 ± 6.2 mmHg to 14.9 ± 3.1 mmHg in the combined Trabectome group. Mean number of medications decreased from 2.9 to 1.2 in the Trabectome group and from 2.6 to 1.5 in the combined group. A total of 14% of patients were considered failure cases from the Trabectome-alone group.

A prospective nonrandomized study grouped open-angle glaucoma patients who underwent Trabectome procedures according to baseline IOP levels [13]. In the group with preoperative IOP levels ≤17 mmHg, the IOP mean reduction was 7% mmHg with a 35% reduction in IOP-lowering medications. However, patients having IOP ≥ 30 mmHg showed IOP reduction as 48% with a 25% reduction in IOP-lowering medications.

Maeda et al. [14] also reported a decrease from mean preoperative IOP of 26.6 ± 8.1 mmHg to 17.4 ± 3.4 mmHg after surgery. The number of IOP-lowering medications decreased from 4.0 ± 1.4 to 2.3 ± 1.2 at 6 months.

In our study, Trabectome surgery with or without cataract surgery achieved fairly good IOP levels from the values of 30 mmHg or higher to mid teens (16.8 ± 3.8). The number of IOP-lowering medications also decreased from 3.1 ± 1.3 to 1.8 ± 1.4 at 12 months.

**Figure 4:**
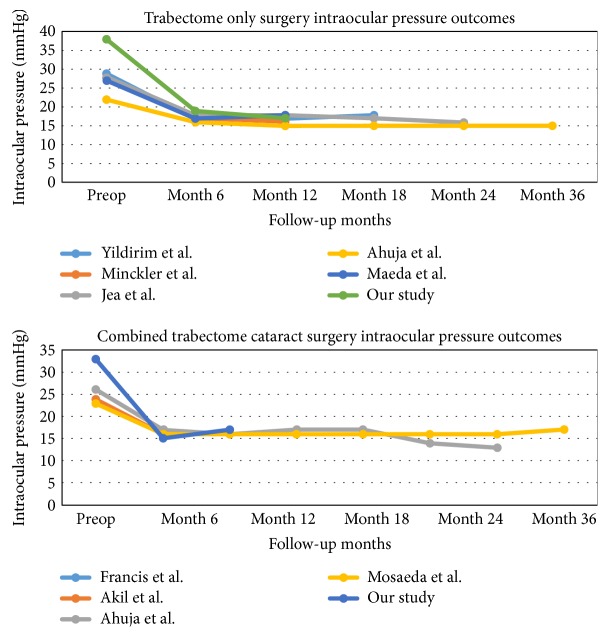
Intraocular pressure (IOP) changes over time after the Trabectome surgery with or without cataract extraction in different studies.

The strengths of our study include having the Trabectome-alone group as controls to determine the IOP-lowering effect of procedures accurately and close monitoring of IOP, medications, and complications in a prospective fashion. Results are presented by differences in mean IOP and glaucoma medications as well as by a Kaplan-Meier survival curve. Our study covers high IOP cases with short-term follow-up; so, it might be valuable to compare the results with the long-term follow-up studies (Figure 4) [4, 6–8, 12, 14–16]. Severe complications like expulsive hemorrhage which may be caused by sudden drop of IOP after the surgery have not been reported yet; therefore, ab interno trabeculotomy using Trabectome might be safer compared to filtration procedures regarding the pressure changes. One of the major limitations of this study is the inclusion of the patients with a high initial IOP (presumably above the mean baseline of all patients undergoing Trabectome). One would anticipate that repeated IOP measurements in this group (even without Trabectome) would be closer to the mean (i.e., lower) on subsequent readings. The other limitations include the nonrandomized design of the study, with the inherent selection bias and dropout issues. Although IOP and a number of medications were found to be lower during follow-up after the surgery, it cannot be claimed that the surgery itself lowered the pressure without a comparison group. Additionally, the patients who maintained a one-year follow-up may have a selection bias. In our study, we did not have a wash-out time interval for glaucoma medications before or after surgery; so, we cannot be certain as to the efficacy or necessity of the number of medications either pre- or posttreatment. We included a comparison group of glaucoma patients who had Trabectome-alone surgery. We encountered some differences between the groups in ethnicity, type of glaucoma, amount of visual field loss, prior surgeries, and degree of angle opening. However, these differences can be expected given the pathogenesis and epidemiology of cataract and glaucoma. The next step would be the establishment of randomized trials to determine the efficacy of Trabectome surgery compared with newer IOP-lowering surgeries for OAG, with one another, and with phacoemulsification alone (in the case of combined procedures).

In conclusion, the risk-to-benefit profile of trabeculotomy by internal approach in patients with high IOP levels has not been studied yet. The results of our study showed that the Trabectome, as a minimally invasive glaucoma surgery, might be considered as an alternative to standard filtration surgery in the surgical treatment of the open-angle glaucoma patients with higher IOP levels because of its internal approach, giving a good option for the combined cataract-glaucoma surgery, the low-risk profile, and the remaining of the future option for filtration surgery.

## Figures and Tables

**Table 1:** Demographics and descriptive statistics of all the patients with IOP ≥ 30 mmHg.

	*n* = 49
Age	
Mean ± SD	66 ± 18
Range	18–91
Gender	
Female	19 (39%)
Male	30 (61%)
Race	
African American	2 (4%)
Asian	5 (10%)
Caucasian	31 (63%)
Hispanics	7 (14%)
Others	4 (8%)
Diagnosis	
POAG	24 (49%)
Pseudoexfoliation glaucoma	12 (24%)
ACG	2 (4%)
Pigment dispersion	5 (10%)
Ocular hypertension	2 (4%)
Secondary glaucoma	2 (4%)
Others	2 (4%)
Preop Snellen acuity	
20/20–20/40	22 (45%)
20/50–20/70	9 (18%)
20/80–20/100	4 (8%)
20/200–20/400	8 (16%)
<20/400	1 (2%)
NR	5 (10%)
VF	
Mild	4 (8%)
Moderate	12 (24%)
Advanced	3 (6%)
MD/others	30 (61%)
Disc C/D	
<0.7	13 (27%)
0.7 to 0.8	17 (35%)
>0.8	11 (22%)
NR	8 (16%)
Lens status	
Phakic	39 (80%)
Pseudophakic	8 (16%)
Aphakic	0 (0%)
NR	2 (4%)
Shaffer grade	
I	0 (0%)
II	2 (4%)
III	11 (22%)
IV	5 (10%)
NR	31 (63%)
Prior surgeries	
SLT	17 (35%)
ALT	4 (8%)
Trabeculectomy	1 (2%)
Trabectome	2 (4%)
YAG	1 (2%)
Combined surgeries	
Trabectome + Phaco	21 (43%)
Trabectome only	28 (57%)

**Table 2:** Demographics and descriptive statistics of the patients with IOP ≥ 30 mmHg and having undergone combined Trabectome surgery.

	*n* = 21
Age	
Mean ± SD	72 ± 17
Range	23–88
Gender	
Female	12 (57%)
Male	9 (43%)
Race	
African American	1 (5%)
Asian	3 (14%)
Caucasian	12 (57%)
Hispanics	5 (24%)
Diagnosis	
POAG	6 (29%)
Pseudoexfoliation glaucoma	9 (43%)
ACG	2 (10%)
Ocular hypertension	1 (5%)
Secondary glaucoma	1 (5%)
Others	2 (10%)
Preop Snellen acuity	
20/20–20/40	5 (24%)
20/50–20/70	6 (29%)
20/80–20/100	3 (14%)
20/200–20/400	6 (29%)
<20/400	0 (0%)
NR	1 (5%)
VF	
Mild	1 (5%)
Moderate	4 (19%)
Advanced	0 (0%)
MD/others	16 (76%)
Disc C/D	
<0.7	5 (24%)
0.7 to 0.8	9 (43%)
>0.8	5 (24%)
NR	2 (10%)
Lens status	
Phakic	20 (95%)
Pseudophakic	0 (0%)
Aphakic	0 (0%)
NR	1 (5%)
Shaffer grade	
I	0 (0%)
II	1 (5%)
III	4 (19%)
IV	1 (5%)
NR	15 (71%)
Prior surgeries	
SLT	9 (43%)
ALT	1 (5%)
Trabeculectomy	1 (5%)

**Table 3:** Demographics and descriptive statistics of the patients with IOP ≥ 30 mmHg and having undergone Trabectome-alone surgery.

	*n* = 28
Age	
Mean ± SD	62 ± 18
Range	30–91
Gender	
Female	7 (25%)
Male	21 (75%)
Race	
African American	1 (4%)
Asian	2 (7%)
Caucasian	19 (68%)
Hispanics	2 (7%)
Other	4 (14%)
Diagnosis	
POAG	18 (64%)
Pseudoexfoliation glaucoma	3 (11%)
Pigment dispersion	5 (18%)
Ocular hypertension	1 (4%)
Secondary glaucoma	1 (4%)
Preop Snellen acuity	
20/20–20/40	17 (61%)
20/50–20/70	3 (11%)
20/80–20/100	1 (4%)
20/200–20/400	2 (7%)
<20/400	1 (4%)
NR	4 (14%)
VF	
Mild	3 (11%)
Moderate	8 (29%)
Advanced	3 (11%)
MD/others	14 (50%)
Disc C/D	
<0.7	8 (29%)
0.7 to 0.8	8 (29%)
>0.8	6 (21%)
NR	6 (21%)
Lens status	
Phakic	19 (68%)
Pseudophakic	8 (29%)
NR	1 (4%)
Shaffer grade	
I	0 (0%)
II	1 (4%)
III	7 (25%)
IV	4 (14%)
NR	16 (57%)
Prior surgeries	
SLT	8 (29%)
ALT	3 (11%)
Trabectome	2 (7%)
YAG	1 (4%)
